# The SOFA score—development, utility and challenges of accurate assessment in clinical trials

**DOI:** 10.1186/s13054-019-2663-7

**Published:** 2019-11-27

**Authors:** Simon Lambden, Pierre Francois Laterre, Mitchell M. Levy, Bruno Francois

**Affiliations:** 1 Department of Medicine, Addenbrooke’s Hospital, University of Cambridge, Cambridge, CB20Q UK; 2 St Luc University Hospital, Université Catholique de Louvain, Avenue Hippocrate 12, 1200 Brussels, Belgium; 3 Rhode Island Hospital, Alpert Medical School, Brown University, Providence, RI USA; 4 Intensive care unit & Inserm CIC 1435 & Inserm UMR 1092, Dupuytren University Hospital, Limoges, France

**Keywords:** Sepsis, Sequential Organ Failure Assessment, SOFA, Clinical Trials, Surrogate endpoints, Critical Care trials, Multiple organ failure

## Abstract

The Sequential Organ Failure Assessment or SOFA score was developed to assess the acute morbidity of critical illness at a population level and has been widely validated as a tool for this purpose across a range of healthcare settings and environments.

In recent years, the SOFA score has become extensively used in a range of other applications. A change in the SOFA score of 2 or more is now a defining characteristic of the sepsis syndrome, and the European Medicines Agency has accepted that a change in the SOFA score is an acceptable surrogate marker of efficacy in exploratory trials of novel therapeutic agents in sepsis. The requirement to detect modest serial changes in a patients’ SOFA score therefore means that increased clarity on how the score should be assessed in different circumstances is required.

This review explores the development of the SOFA score, its applications and the challenges associated with measurement. In addition, it proposes guidance designed to facilitate the consistent and valid assessment of the score in multicentre sepsis trials involving novel therapeutic agents or interventions.

Conclusion

The SOFA score is an increasingly important tool in defining both the clinical condition of the individual patient and the response to therapies in the context of clinical trials. Standardisation between different assessors in widespread centres is key to detecting response to treatment if the SOFA score is to be used as an outcome in sepsis clinical trials.

## Background

The SOFA score has become integrated into a range of aspects of critical care since its development in the early 1990s, and it is now widely employed in the daily monitoring of acute morbidity in critical care units. The SOFA score was designed to provide population level insights into the acute morbidity of ICU patients; however, its application has broadened substantially in recent years. Following the development of new definitions [1–3], it is now used as a key criterion in the diagnosis of the sepsis syndrome on an individual patient level [3]. It is also increasingly used to determine the efficacy of novel therapeutic agents in phase II trials, a development that follows acceptance by the European Medicines Agency (EMA) and others of organ dysfunction scores as an endpoint in exploratory trials for sepsis [4].

This review describes the development of the score and the challenges associated with robust and reproducible calculation and proposes guidance for its assessment in clinical trials, where inconsistency in SOFA score measurement could introduce substantial variability in key outcomes.

### The development of the SOFA score

The SOFA (Sequential Organ Failure Assessment) score was developed following a consensus meeting in 1994, the stated aim of which was to create a score ‘to describe quantitively and as objectively as possible the degree of organ dysfunction/failure over time in groups of patients or even individual patients’ [5]*.* The score was designed to describe a sequence of complications of critical illness and not to predict outcome, although the authors acknowledged that any functional morbidity score must also be associated with mortality. Initially described as the sepsis-related organ failure assessment, the utility of the score for the assessment of acute morbidity in a range of critical illnesses was recognised early and the title changed.

SOFA was based on six different scores, one for each of the respiratory, cardiovascular, hepatic, coagulation, renal and neurological systems each scored from 0 to 4 with an increasing score reflecting worsening organ dysfunction [5, 6]. The development team showed retrospectively that the score detected differences in severity of illness [5] and proposed its use as an alternative to other assessments of multiple organ dysfunction that had been developed in the early 1990s [7].

Following its initial validation, prospective analysis of the score’s utility was undertaken in 16 countries [6]. The study showed that some sub-scores and also the total score were associated with survival. Moreno et al. [8] studied the impact of maximum SOFA score in the same population and showed that there was a good correlation between increasing score and mortality. The score performed well as a discriminator of survival status at ICU discharge. In addition to studying the maximum SOFA score, the change in score, or delta SOFA (total maximum SOFA score minus admission total SOFA score) also demonstrated a strong correlation with ICU mortality.

Further prospective evaluations in differing settings have validated the SOFA score, its maximum value during ICU stay and also change in SOFA over time as valid tools for the assessment of morbidity in critical illness [9–12], and the score has become a common feature of observational study reporting.

### Calculation of the SOFA score standard approach

SOFA score may traditionally be calculated on admission to ICU and at each 24-h period that follows. The tool employs six criteria reflecting the function of an organ system (respiratory, cardiovascular, renal, neurological, hepatic and haematological) and allocates a score of 0–4 as described below in Table 1.

**Table 1:** The criteria for assessment of the Sequential Organ Failure Assessment (SOFA) score

Respiratory system	
PaO_2_/FiO_2_ (mmHg)	SOFA score
> 400	0
< 400	1
< 300	2
< 200 with respiratory support	3
< 100 with respiratory support	4
Nervous system	
Glasgow Coma Scale	SOFA score
15	0
13–14	1
10–12	2
6–9	3
< 6	4
Cardiovascular system	
Mean arterial pressure (MAP) OR administration of vasopressors required	SOFA score
MAP > 70 mmHg	0
MAP < 70 mm/Hg	1
Dopamine ≤ 5 μg/kg/min or dobutamine (any dose)	2
Dopamine > 5 μg/kg/min OR epinephrine ≤ 0.1 μg/kg/min OR norepinephrine ≤ 0.1 μg/kg/min	3
Dopamine > 15 μh/kg/min OR epinephrine > 0.1 μg/kg/min OR norepinephrine > 0.1 μg/kg/min	4
Liver	
Bilirubin (mg/dl) [μmol/L]	SOFA score
< 1.2 (< 20)	0
1.2–1.9 [20–32]	1
2.0–5.9 [33–101]	2
6.0–11.9 [102–204]	3
> 12.0 [> 204]	4
Coagulation	
Platelets ×10^3^/ml	SOFA score
> 150	0
< 150	1
< 100	2
< 50	3
< 20	4
Kidneys	
Creatinine (mg/dl) [μmol/L]; urine output	SOFA score
< 1.2 [< 110]	0
1.2–1.9 [110–170]	1
2.0–3.4 [171–299]	2
3.5–4.9 [300–440] (or urine output < 500 ml/day)	3
> 5.0 [> 440]; urine output < 200 ml/day	4

Modified from Vincent et al. [5]

In cases where the physiological parameters do not match any row, zero points are given. In cases where the physiological parameters match more than one row, the row representing the highest score is selected.

### SOFA score terminology

The SOFA score has been applied in a range of applications with some variation in the terminology employed. A number of terms are commonly used and are associated with the following definitions:

- Admission SOFA: The admission SOFA score is calculated based on the most severe value for each sub-score in the 24 h preceding admission to ICU [9].
- Daily Maximum SOFA score: The daily maximum SOFA score is equivalent to the daily SOFA score as when calculated for each 24 h assessment; the most severe value of each sub-score for that time period should be calculated in the assessment of the SOFA score.
- Maximum SOFA score: The maximum SOFA score describes the highest daily SOFA score over the course of the study period.
- Delta SOFA score: The delta SOFA is calculated as the change in total SOFA score (or that of an individual sub-score) between a defined time point and the baseline value. The baseline value may be the admission SOFA or a defined study day.
- Mean SOFA: The mean SOFA score is calculated for an individual patient over the course of a defined study period based on the total SOFA score for each study day.

### Generic rules for measuring components of the SOFA score

A number of standard rules have been proposed for the calculation of SOFA score values [9] .

#### Selecting the daily value

The value for each sub-score that represents the most severe (worst) value for the respective 24-h period for each parameter was used in initial validation and subsequent clinical studies using the SOFA score.

##### Proposal 1

SOFA score should be undertaken prior to the start of any intervention or admission and for each subsequent 24-h period. At each assessment, the worst (most severe) value for the 24-h period of each SOFA sub-score is selected.

##### Proposal 2

If data points arise in more than one score for a subcategory, the higher SOFA sub-score criteria is selected.

#### Handling missing data

In their initial development of the SOFA score, Vincent et al. [5] dealt with a single missing value by calculating a replacement from the mean of the sum of the values immediately preceding the missing value. Moreno et al. used the mean of the preceding and immediately succeeding values [6, 8], with two consecutive missing results leading to the value be treated as a missing data point. Other groups have used the last observation carried forward (LOCF) approach in the event of missing values [13], although this approach will not be effective for data missing on the first study day, and how this possibility may be handled using methods such as carrying back a succeeding value or using the pre-randomisation score should be considered.

In the event of death during the assessment period, data for some patients, many of whom will have high scores, will be missing, leading to a survivorship bias which may paradoxically favour the study group with higher mortality. As such, it is essential for study teams to include robust rules for handling this eventuality. Teams could consider a range of approaches to this issue. The first of these include imputation of the last recorded value for the total or individual sub-score. This will provide a ‘complete’ data set for analysis; however, it does not account in any way for patients who do not survive. A second strategy is to apply a maximum sub- or total value for patients who do not survive to the end of the SOFA assessment period. This approach means that the association of higher SOFA score with outcome will be preserved in subsequent analyses and the result is protected from missing data but does not directly account for early mortality. A third strategy to account for early mortality is to ascribe an additional penalty in the event of death during the SOFA assessment period. This additional penalty ensures that early mortality is ‘included’ in the SOFA assessment in addition to acute morbidity. To date, no consensus has been achieved in how the issue of missing data due to death should be handled. The importance of this issue has been recently highlighted in the CITRIS-ALI trial of vitamin C in patients with sepsis-associated acute lung injury. In their study, Fowler et al. demonstrated a reduction in the unadjusted secondary outcome of mortality without apparent trend in the primary outcome, the change in a modified SOFA score [14]. In the absence of an imputed score or penalty for death, patients that did not survive were removed from the analysis meaning that a differential impact on delta SOFA may not have been detected.

It is important to recognise that in clinical trials, imputation of missing data introduces risks of bias due to the nature of the missing data and the way it is handled. Detailed examination of this is beyond the scope of this review; however, data is considered missing completely at random (MCAR) if the missing data arises as a consequence of neither the observed nor the missing data. Missing at random (MAR) data depends only on the observed data, and missing not at random (MNAR) data arises if the mechanism depends on the missing data; this dependency remains even given the observed values. MAR data may be imputed or handled using other methods without the introduction of systematic bias; however, if MNAR data is present, this may not be possible [15]. A range of sensitivity analyses are available to determine the nature of the missing data and should be included in the statistical analysis plan for any randomised controlled trial [16].

##### Proposal 1

In a clinical trial that employs SOFA score as a primary or key secondary outcome, centres should conduct laboratory measurement of the relevant SOFA variables daily if possible.

##### Proposal 2

In the event of a missing value, study teams should define their approach to missing data a priori*.* Possible methods include the mean of the preceding and immediately succeeding values or last observation carried forward. The use of this approach should only apply to a single missing value and should not be used to impute missing data from two or more days.

##### Proposal 3

In patients included in randomised controlled trials, a priori rules should be established for calculating SOFA score and sub-scores in the event of death prior to the end of the period of SOFA recording.

### The central nervous system (CNS) SOFA component

The CNS component of the SOFA score is the least accurately measured and associated with the most errors [17]. In their initial validations, the Vincent group used an assumed value for the Glasgow Coma Scale (GCS) in patients receiving sedation [5, 6, 9] which is associated with significant variability in the recorded value [17]. Other studies have employed a method where last GCS recorded prior to intubation is carried forward in the daily assessment until the patient can be examined neurologically in the absence of sedation. If no value is recorded prior to intubation, then a normal (GCS 15/15) value is often inferred [18]. Modifications to the SOFA score to mitigate this variability have been proposed and are addressed below.

Limited evidence exists for the optimum delay before reliable assessment of GCS can be made after the hypnotic medication is stopped. In cases where confidence that clearance of sedative agents is complete is essential such as brain stem death testing, a delay of up to four times the elimination half-life of the treating agent is considered the standard in some countries [19]. In the context of SOFA scoring in clinical trials however, this amount of time is unlikely to be necessary in all cases, and a pragmatic assessment must be made. In clinical trials, consistency of assessment across centres and assessors is vital; therefore, design of clinical trial protocols should include assessment rules that minimise the risk of variability.

#### Proposal 1

The GCS value will be carried over from the last pre-intubation GCS throughout the duration of hypnotic/sedative medication administration.

if:

GCS from before intubation is not available, a value of 15/15 will be recorded and carried over throughout the duration of hypnotic/sedative medication administration.

#### Proposal 2

Formal assessment of GCS can be undertaken from 24 h after the cessation of sedative medication by infusion.

if:

The clinician at the bedside is satisfied that the assessment is not affected by ongoing effects of sedative/hypnotic therapy.

#### Proposal 3

In clinical trials, GCS assessment training should be undertaken by those with responsibility for formal SOFA scoring. This is of particular relevance if values are extracted from electronically recorded patient data.

### The respiratory SOFA component

Assessment of the respiratory SOFA score relies on invasive arterial monitoring to measure arterial partial pressure of oxygen followed by calculation of the PaO_2_/FiO_2_ ratio. This assessment may prove challenging when arterial monitoring is not employed. Some studies have developed tools to facilitate calculation of a respiratory SOFA component based on peripheral arterial saturations [20], although there is not sufficient evidence base to recommend this approach at this stage.

In addition to fixed performance (venturi) oxygen masks, many patients will be treated at some stage in their care with conventional nasal cannula, standard facemasks or a mask with reservoir bag, all of which deliver oxygen at variable flow rates and inspired oxygen percentage. An approximation of the FiO_2_ associated with their use may be employed for SOFA score calculation [20]. For patients on nasal cannula oxygen, an estimated FiO_2_ may be calculated by multiplying the litre flow/minute by 0.03 and adding that to 0.21 (Table 2) [20]. Estimation of FiO_2_ in patients receiving supplementary oxygen via facemask (without venturi device) or facemask with a reservoir bag should be derived from Table 3 [21].

**Table 2:** Estimated FiO_2_ in patients receiving ventilatory support using simple nasal cannula

Estimated FiO_2_ in patients supported with low flow nasal cannula								
Flow rate (l/min)	1	2	3	4	5	6	7	8
Estimated FiO_2_	0.24	0.27	0.3	0.33	0.36	0.39	0.42	0.45

Adapted from Sedangire et al. [20]

**Table 3:** Estimated FiO_2_ in patients receiving ventilatory support using facemasks

Estimated FiO_2_ in patients supported with oxygen via facemask				Estimated FiO_2_ in patients supported with oxygen via facemask with reservoir bag					
Flow rate (l/min)	5	6–7	7–8	Flow rate (l/min)	6	7	8	9	10+
Estimated FiO_2_	0.4	0.5	0.6	Estimated FiO_2_	0.6	0.7	0.8	0.9	0.95

Adapted from the International study of the prevalence and outcomes of infection in intensive care units [21]

The SOFA score calls for patients to receive a score of 3 or 4 if they reach a PaO_2_/FiO_2_ ratio of less than 200 or less than 100 respectively and are receiving respiratory support. In addition to invasive and non-invasive ventilators, high flow rate oxygen delivered at a controlled percentage via a dedicated nasal cannula has become more prevalent in the years since the development of the SOFA score. These devices are reported to offer a fixed delivered oxygen percentage and a degree of positive end expiratory pressure (PEEP), although the true inspired concentration and amount of PEEP delivered is dependent on the flow rate and a number of patient factors and does not exceed 5 cmH_2_O [22].

#### Proposal 1

The PaO2/FiO2 ratio will be calculated for all patients with an indwelling arterial cannula for any part of each day and the lowest value for that 24-h period used to calculate the respiratory SOFA score.

#### Proposal 2

For patients on nasal cannula oxygen, an estimated FiO_2_ may be calculated by multiplying the litre flow/minute by 0.03 and adding that to 0.21 or using a standard table.

#### Proposal 3

Patients dependent upon high flow nasal cannula (HFNC) to maintain adequate oxygenation should have their PaO_2_/FiO_2_ ratio calculated based on the fraction of inspired oxygen set by the device.

### The cardiovascular (CVS) SOFA component

The existing standard SOFA characteristics include a standard value for the use of dopamine, dobutamine, epinephrine or norepinephrine. It is now common in clinical practice to add vasopressin (ADH) and its analogues to the management of septic shock as part of the standard of sepsis care to reduce norepinephrine dose required to achieve a target MAP [23]. Additional vasopressor agents such as terlipressin and angiotensin II may be used in some centres and may have a norepinephrine sparing effect although formal evidence of their dose equivalence with norepinephrine is lacking; therefore, agents should be considered when calculating an equivalent norepinephrine dose.

The conversion table below (Table 4) is derived from a number of sources [24] and allows study teams to include the dose of vasopressin and other agents as part of the SOFA calculation in order to avoid falsely low CVS SOFA values in patients receiving combination therapy.

**Table 4:** Guidance for the conversion of vasopressor doses in the calculation of the cardiovascular SOFA component

Drug	Dose	Norepinephrine equivalent
Epinephrine	0.1 μg/kg/min	0.1 μg/kg/min
Norepinephrine	0.1 μg/kg/min	0.1 μg/kg/min
Dopamine	15 μg/kg/min	0.1 μg/kg/min
Phenylephrine	1.0 μg/kg/min	0.1 μg/kg/min
Vasopressin	0.04 U/min	0.1 μg/kg/min

Adapted from the protocol for Khanna et al. [24] and Vincent et al. [5] and Liu et al. [25]

The use of defined blood pressure targets can, to some degree confound the calculation of CVS SOFA based on vasopressor dose alone; however, in clinical trials with defined haemodynamic targets, consistency across the study groups should allow robust comparison of the CVS SOFA scores based on the guidance offered below as between group differences in vasopressor requirement will be reflected in the SOFA calculation.

#### Proposal 1

Study teams should define the duration of a period without vasopressor administration that should elapse before an episode of vasopressor therapy is considered complete. Receipt of a vasopressor at any point within the 24-h window of assessment of the SOFA score should merit a score representing that requirement.

#### Proposal 2

Vasopressin may be used as a second agent to reduce total noradrenaline dose. However, the dose of vasopressin used should be converted to an equivalent norepinephrine and the ‘total equivalent norepinephrine dose’ used to determine the CVS SOFA component.

#### Proposal 3

The peak level of cardiovascular support for a given 24-h period should be used to calculate the daily cardiovascular SOFA score.

### The renal SOFA component

The surviving sepsis guidelines call for the use of renal replacement therapy (RRT) in the management of symptomatic renal failure or fluid balance in patients with haemodynamic instability [23]. The SOFA score is based on the clinical indices of creatinine or urine output, both of which will be affected by the presence of renal replacement therapy. Given the wide variety of application of renal replacement therapy between ICUs, this could introduce substantial variability in the SOFA score for patients included in clinical trials. One approach to this would be to consider applying a renal sub-score of four in patients undergoing renal replacement therapy. The period of time that should elapse after cessation of RRT before a patient is considered to have been liberated from renal support is not defined by the literature.

#### Proposal 1

Study teams should develop a formal strategy for SOFA score calculation in patients undergoing renal replacement therapy if using the SOFA score as a key outcome.

### The coagulation SOFA component

The haematology component of the SOFA score is calculated using the measured platelet concentration. The administration of platelet transfusion is not recorded during scoring but may have a significant impact on the measured platelet concentrations and therefore the coagulation component of the SOFA score. Standard guidance from the surviving sepsis council exists for the management of platelet therapy in patients with sepsis [23].

#### Proposal 1

The lowest platelet value for the preceding 24 h should be determined before transfusion (if given), and if platelets are given regularly, the lowest pre-transfusion value should be used to calculate each daily score.

### Improving inter-rater reliability in SOFA assessment

Any score that is dependent upon the assessment of clinical criteria and laboratory variables may be subject to variation in that assessment. Reasons for this include different laboratory assays, changes in personnel undertaking examinations and confounders not measured within the score.

The calculation of the SOFA score is at risk of each of these potential pitfalls. In their 2009 study, Tallgren et al. examined the accuracy of SOFA scoring in a single centre and determined that assessment of the cardiovascular, renal, haematological and liver sub-scores was highly accurate with more than 80% of assessments correct. The respiratory score was correct in 75% of measurements; however, the neurological score was accurate in only 70% of cases. This inconsistency between clinicians meant that only 48% of SOFA scores were fully in agreement with gold standard assessment and a mean difference of 0.66 points existed between actual and gold standard overall SOFA measurement, a degree of variability that is potentially important in determining morbidity [17]. Of note is that expert raters of the SOFA score achieved high degrees of inter-rater consistency across all SOFA sub-scores. The pattern of these data was consistent with an earlier single-centre study of 30 patients, assessed by 20 clinicians [26].

The Finnish study demonstrated that a short training session led to substantial improvements in scoring performance, a reduction in the degree of variation in the overall score and in the number of errors in the overall score greater than one or two points [17].

#### Proposal 1

Studies including SOFA scoring as an inclusion criteria or outcome should consider a formal training package for recruiting centres to reduce inaccuracy and variability in different centres.

### Modified SOFA scores

A number of modifications have been proposed to the SOFA score including assessments that require fewer laboratory measurements. A number of studies have shown that various components of the score can be removed or replaced by using for example, clinical assessment of jaundice rather than serum bilirubin or urine output instead of creatinine. The revised respiratory sub-score using peripheral oxygen saturations discussed above produced results consistent with the standard SOFA assessment [20, 27, 28]. Other approaches include the addition of a further factor such as the time since last infection which offers increased predictive ability in specific patient groups, for example in populations with haematological malignancy [29, 30].

It has been proposed that the neurological component of the SOFA score could be replaced with an alternative measure such as the Richmond Agitation and Sedation Score (RASS) [31]; however, since the RASS is a marker of sedation and not neurological status, this approach has not been recommended as an approach by the original developers of the SOFA score [32]. An alternative is that the neurological sub-score could be removed to produce a five-component modified SOFA (mSOFA) [33] This approach has proven to be valid and produced results consistent with the use of GCS to calculate the CNS component of the score [13].

In small studies in specific or centres or environments, modified SOFA scoring may offer an attractive solution to some of the challenges of standard SOFA. However, these tools have not been validated prospectively across multiple centres and therefore cannot be recommended as replacement for the traditional approach at this stage. In addition, some of these scores potentially increase the likelihood of inaccuracy due to a reduction in the number of laboratory assays that they employ and dependence on clinical assessment by individuals.

### Extending the application of SOFA scoring

#### Defining sepsis

Defining the syndrome of sepsis has proven challenging since the initial consensus definitions were developed in the early 1990s [34]. The definitions of sepsis and septic shock were based on expert consensus [35–38]. In 2016, a novel approach saw a data-driven redefinition as:

‘Life threatening organ dysfunction caused by a dysregulated host response to infection’ [3].

The team demonstrated that SOFA score was a better discriminant than the traditional SIRS and similarly effective to the more complex Logistic Organ Dysfunction System (LODS) [1]. Organ dysfunction was therefore characterised by a change in SOFA score of two or more points as a consequence of infection, which conferred an associated mortality of approximately 10%. By using a change in SOFA score, the authors recognised that whilst SOFA score can often be considered zero in previously healthy patients, the presence of chronic organ dysfunction precludes the use of an absolute value to define the presence of infection [3]. This transition from observing to defining a syndrome has significant relevance for clinicians and researchers in critical care.

#### Using SOFA as an outcome in clinical trials

The association of SOFA score at admission and during ICU stay with long-term outcomes has led a number of investigators to propose SOFA or delta SOFA as a potentially valid surrogate in clinical trials. This approach confers the advantage that shorter periods of follow-up are required to determine efficacy, although this is valid only if a change in SOFA is a clinically relevant outcome or that is a true surrogate of a later important outcome. This approach will have greater validity if, as with all composite outcomes, study teams also report the sub-scores that make up the SOFA as part of the trial data.

In the ATHOS-3 trial [24], a key secondary end point was a change in the CVS SOFA score which displayed a significant improvement over the study period in patients treated with angiotensin II. Interestingly, the study did not calculate vasopressor dose equivalence in the intervention group including angiotensin II, a limitation that future studies of vasopressors should consider addressing.

In contrast, the upcoming STRESS-L study of the impact of treatment with the beta blocker Landiolol will use ‘the mean SOFA score over the first 14 days from entry to the trial and whilst in ICU’ as the primary outcome measure in patients with septic shock and a noradrenaline requirement of ≥ 0.1 μg/kg/min [39]. This approach confers the advantage that in the event of a patient death prior to the end of study, the mean SOFA score over the period remains comparable across all patients regardless of duration of survival and means that no patients are excluded from the end point analysis.

de Grooth et al. [40] interrogated the use of SOFA and its association with mortality in 87 studies. They looked at the relationship between using a SOFA at a defined time point in the study (fixed day SOFA) which allows comparison of acute morbidity at a defined time point across study groups and delta SOFA (which was defined as the change in SOFA score from baseline/maximum to a defined time point). They demonstrated that using delta SOFA was significantly correlated with mortality with a low degree of heterogeneity. A fixed day SOFA as an endpoint was not reliably associated with mortality. The authors note that many of the included studies were small (median (IQR) 64(40–147) patients).

## Discussion

The SOFA score was developed to describe the acute morbidity of patient populations with critical illness in different settings. The use of the tool for this purpose has been repeatedly validated and, over the years that followed its development, its role has extended to a range of new indications. It is now a defining characteristic of the sepsis syndrome which means that interventions and treatments delivered to individual patients depend on precise and consistent assessment of the score. In addition, the acceptance by the EMA that in exploratory clinical trials in sepsis, a change in organ dysfunction scores is a valid endpoint [4], has led to the change in SOFA score being selected as a primary outcome in a number of recent and ongoing studies, alongside the reporting of mortality .

There is evidence from a range of observational study settings that even a modest change in SOFA score is associated with a persistent trend in mortality. This includes a change in SOFA between ICU and ED admission [41] at 48 h in sepsis associated disseminated intravascular coagulation [42], following cardiac arrest [43] and in general critical illness [44] as well as at day 7 in pancreatitis [45].

In the context of randomised trials, de Grooth et al. identified 25 studies where the change in SOFA score from baseline or maximum to a defined time point was used and revealed a strong association between change in SOFA and mortality (*p* = 0.004), with 32% of the observed mortality effects explained by the delta SOFA [40]. They went on to recommend, based on the mean standard deviation of those studies, that 110 patients would be required in each treatment arm of a study to detect a one point difference in delta SOFA. If detected, they inferred that this would in turn be associated with a mortality odds ratio of 2. The authors concluded that aiming to detect a greater difference than this would be unrealistic and therefore this should represent a minimum sample size in studies using delta SOFA as a primary endpoint. It is important to recognise therefore that the ability to detect single-integer changes in the overall SOFA score with low inter-individual and inter-centre variability becomes essential in the conduct of randomised trials employing this outcome.

Like all scores that assess the clinical course of critically ill patients based at least in part upon levels of organ support and assessments undertaken at single time points, SOFA scores can, as we describe, be confounded by clinical interventions. As a consequence, the development of standard protocols for the assessment and management of patients in clinical trials is essential in order to minimise inter-patient variability and ensure that results of surrogate assessments like SOFA are robust.

## Conclusion

In this review, we propose solutions and pragmatic approaches to calculating the SOFA score which have the potential to improve the reliability of assessments and mitigate some of the sources of heterogeneity that could prove important in new applications of the score. Training of study teams in the measurement of the SOFA score and application of study guidance is an important part of this process and should be considered in all studies including the SOFA score as an inclusion criteria or end point. The evidence base available to determine the guidance presented here is limited, and study authors should consider this before defining the approaches they will take to assessment of the SOFA score. Balancing the requirement for robust and consistent calculation with the introduction of unvalidated approaches and the inadvertent development of a new scoring system is an important challenge for clinical triallists to address.

## Data Availability

NA

## Abbreviations

CNS: Central nervous system
CVS: Cardiovascular system
EMA: European Medicines Agency
FiO_2_: Fraction of inspired oxygen
GCS: Glasgow Coma Scale
HFNC: High flow nasal cannulae
ICU: Intensive care unit
IQR: Interquartile range
LOCF: Last observation carried forward
LODS: Logistic Organ Dysfunction Score
MAP: Mean arterial pressure
PaO_2_: Partial arterial pressure of oxygen
PEEP: Positive end expiratory pressure
RASS: Richmond Agitation and Sedation Score
RRT: Renal replacement therapy
SOFA: Sequential Organ Failure Assessment
SpO_2_: Peripheral oxygen saturation
